# Early-life stress differentially affects CA3 synaptic inputs converging on apical and basal dendrites of CA1 pyramidal neurons

**DOI:** 10.3389/fncir.2025.1533791

**Published:** 2025-02-19

**Authors:** David Jappy, Rostislav Sokolov, Yulia Dobryakova, Viktoriya Krut’, Ksenia Maltseva, Anastasia Fedulina, Ivan Smirnov, Andrei Rozov

**Affiliations:** ^1^Federal Center of Brain Research and Neurotechnologies, Moscow, Russia; ^2^Institute of Neuroscience, Lobachevsky State University of Nizhniy Novgorod, Nizhny Novgorod, Russia; ^3^Laboratory of Neurobiology, Kazan Federal University, Kazan, Russia

**Keywords:** early life stress, LTP, hippocampus, excitation, CA1 pyramidal cell

## Abstract

There is evidence that stress factors and negative experiences in early in life may affect brain development leading to mental disorders in adulthood. At the early stage of postnatal ontogenesis, the central nervous system has high plasticity, which decreases with maturation. Most likely, this high plasticity is necessary for establishing synaptic connections between different types of neurons, regulating the strength of individual synapses, and ultimately forming properly functioning neuronal networks. The vast majority of studies have examined the effects of early-life stress (ELS) on gene expression or behavior and memory. However, the impact of ELS on functional synaptic development and on the plastic properties of excitatory and inhibitory synapses are currently much less understood. Based on data obtained in a few studies it has been suggested that ELS reduces long-term potentiation (LTP) at Schaffer collateral to CA1 pyramidal cell synapses in adulthood. Nevertheless, different groups have reported somewhat contradictory results. In this report we show that ELS differentially affects LTP at CA3 to CA1 pyramidal cell inputs, at synapses on apical dendrites LTP is reduced, while LTP at synapses formed by CA3 pyramidal cells on basal dendrites remains unaffected.

## Introduction

There is evidence that adverse early life experiences can lead to changes in brain development and mental disorders in adulthood (Heim and Nemeroff, 2002). It has been reported that early-life stress (ELS) induced by providing limited bedding and nesting material to female mice and their offspring during the period from the 2nd to the 9th day after birth reduces the expression of NMDA receptors and leads to the occlusion of CA1 LTP (Lesuis et al., 2019) selectively in adult males. However, a more recent study shows that ELS selectively enhanced NMDAR function in adolescent female mice and had no effect on LTP regardless of the sex of the animals (Wilkinson et al., 2024). ELS induction protocols can also have opposing effects on hippocampal plasticity. It has been shown that maternal separation results in decreased Schaffer collateral LTP (Herpfer et al., 2012; Sousa et al., 2014). However, Ryan et al. (2009) published data suggesting that maternal separation caused both increased α-amino-3-hydroxy-5-methyl-4-isoxazolepropionic acid receptor (AMPAR) and *N*-methyl-D-aspartate receptor (NMDAR) expression and increased LTP in rat CA1 pyramidal cells. At cortical inputs to DG granule cells maternal deprivation results in increased LTP (Kehoe et al., 1995; Bronzino et al., 1996; Kehoe and Bronzino, 1999; Blaise et al., 2008), while maternal separation had no effect on LTP (Oomen et al., 2010; Oomen et al., 2011).

In most studies characterizing the effects of ELS on NMDA-dependent LTP in CA1 pyramidal cells, plasticity was induced by either high-frequency (100 Hz) stimulation or theta-burst stimulation. Both protocols are widely used in plasticity studies, however, in addition to triggering NMDA receptor-mediated calcium influx, which should be sufficient to induce LTP at these synapses, these protocols can trigger action potential firing in the postsynaptic cell and hence activation of dendritic voltage-gated calcium channels. The latter results in an additional increase in cytosolic Ca^2+^ levels throughout the dendritic tree. Theta burst stimulation might be more physiological, but the additional calcium influx may compensate for the possible functional deficit caused by ELS, which could otherwise lead to altered plasticity.

Another issue that has been overlooked in ELS studies is the synapse specificity of possible LTP changes. It has been shown that in approximately 25% of hippocampal pyramidal cells, the axon originates from one of the basal dendrites, making the CA3 to CA1 projections in *stratum oriens* functionally distinct from the *stratum radiatum* inputs (Thome et al., 2014; Hodapp et al., 2022).

In this study, we tested the effects of maternal separation on pairing-induced LTP at synapses between CA3 and CA1 pyramidal cells in the ventral hippocampus of adolescent (P35-49) mice. The synaptic properties and LTP magnitude at synapses converging on apical and basal dendrites were analyzed separately. Data and analyses were also stratified by animal sex. We found that in CA1 pyramidal cells, maternal separation significantly and selectively reduced LTP at excitatory synapses on apical dendrites. Plastic properties at excitatory inputs to basal dendrites from CA3 neurons were unaffected by ELS. To confirm our findings that maternal separation reduces LTP levels at synapses located on apical dendrites of CA1 pyramidal cells, we performed *in vivo* experiments in rats. We found that ELS suppresses LTP at the apical excitatory inputs to CA1 pyramidal cells in the dorsal hippocampus of adult rats, regardless of animal sex.

## Materials and methods

### Study design

**FIGURE 1:**
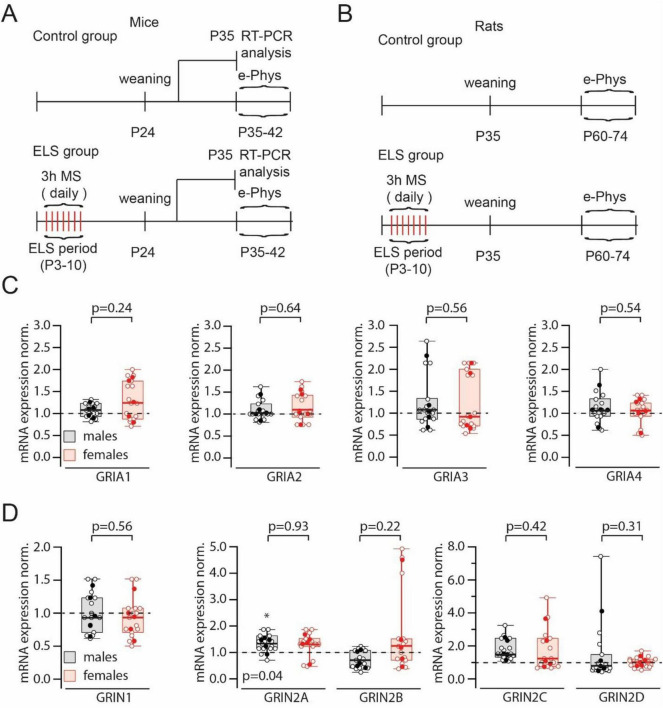
Experimental design and results of iGluR expression analysis. **(A)**. The diagram shows the experiment design for mice: the timeline of maternal separation sessions (MS), the date of taking hippocampal tissue for RT-PCR analysis and the period when animals were used for electrophysiological experiments. **(B)**. The diagram shows the experiment design for rats: the timeline of MS and the period when animals were used for electrophysiological experiments. **(C)**. Comparison of normalized to control (control mice) GRIA1–GRIA4 mRNAs levels in early-life stress (ELS) males and females. Data on box plots are presented as the median (P_25_; P_75_). Whiskers show minimum and maximum values. The statistical significance of the differences between two genders was assessed using the Mann-Whitney Rank Sum Test. Relative to control animals the levels of mRNA for all four subunits in ELS mice were not significantly different (*p* > 0.05; Mann-Whitney Rank Sum Test). Open symbols represent data from RT-PCR runs; filled symbols show the averaged value for each animal. **(D)**. Comparison of normalized to control (control mice) GRIN1 (left plot), GRIN2A and B (middle plot) and GRIN2C and D (right plot) mRNAs levels in ELS males and females. Data on box plots are presented as the median (P_25_; P_75_). Whiskers show minimum and maximum values. The statistical significance of the differences between two genders were assessed using the Mann-Whitney Rank Sum Test. The levels of mRNA for GRIN1, GRIN2B, GRIN2C and GRIN2D subunits in ELS mice relative to control animals were not significantly different (*p* > 0.05; Mann-Whitney Rank Sum Test). Note the small but significant (*) elevation of GRIN2A mRNA in ELS males. Open symbols represent data from RT-PCR runs; filled symbols show the averaged value for each animal.

All experiments were conducted on C57BL/6J mice and Wistar rats. Control mice and animals for the early-life stress group were bred in the same vivarium. Control dams and their pups were kept under standard conditions. After weaning on day 24, the offspring were separated by sex. On the 35*^th^* day five males and five females were sacrificed for RT-PCR analysis of GluR expression. Animals were also used for electrophysiological experiments starting from day 35 after birth. Early life stress (ELS) was induced by daily separation from the mother, and the pups were placed in individual cages for 3 h from day 3 to day 10 after birth (Figure 1A).

*In vivo* experiments were performed on Wistar rats, the housing conditions and the ELS protocol were identical to those described above. Electrophysiological experiments were performed when the animals reached the age of 35 days (Figure 1A).

### Early life stress

All experimental protocols were approved by the Local Ethical Committee of Kazan Federal University (#24/22.09.2020). Control mouse (C57BL6/J) or rat (Wistar) litters were maintained undisturbed in their home-cages until weaning. ELS was caused by maternal separation, pups were removed from their cages daily as of postnatal day (P) 3 to P10 for 180 min and placed in an isolated cage without bedding material and additional heating. At the end of the separation period, pups were returned to their home-cage. No changes in body weight or size were observed between control and ELS animals.

### RT-PCR analysis

The total RNA was isolated from the dissociated hippocampus of males and females of the C57Bl/6J mouse line using the Extract RNA reagent (Evrogen, Russia). Reverse transcription and RT-PCR were performed using the One Tube RT-PCR SYBR kit (Evrogen, Russia) using a Rotor Gene 6 Plex amplifier (Qiagene, Germany). The samples were set up in three repeats using the following reaction protocol: 55°C for 15 min, then 95°C for 1 min and 40 cycles of: denaturation (15 s at 95°C), annealing (20 s at 60°C) and elongation (20 s at 72°C). The primers used for glutamate ionotropic AMPA receptor (GRIA) and glutamate ionotropic NMDA receptor (GRIN) (Priya et al., 2014; Imbriglio et al., 2021) are shown in the Table 1.

**TABLE 1:** List of the primers used for RT-PRC analysis.

Name	Forward primer	Reverse primer
GRIA1	5′-GAGCAACGAAAGCCCT GTGA-3′	5′-CCCTTGGGTGTCGC AATG-3′
GRIA2	5′-AAAGAATACCCTGGAG CACAC-3′	5′-CCAAACAATCTCCT GCATTTCC-3′
GRIA3	5′-TTCGGAAGTCCAAGGGA AAGT-3′	5′-CACGGCTTTCTCTG CTCAATG-3′
GRIA4	5′-GGCTCGTGTCCGCA AGTC-3′	5′-TTCGCTGCTCAATGTAT TCATTC-3′
GRIN1	5′-AACCTGCAGCAGTACC ATCC-3′	5′-GCAGCAGGACTCATCA GTGT-3′
GRIN2A	5′-TCTCCGCCTTTCCGAT TTGG-3′	5′-TGGCAAAGATGTACCC GCTC-3′
GRIN2B	5′-CGCTCTCCACACCCTG AGAT-3′	5′-TAGAAGCCAAAGCTCT AGGC-3′
GRIN2C	5′-CATTAGGGATTTCCCC AAACGC-3′	5′-ACCTTCCTAGTCCA AGCACA-3′
GRIN2D	5′-TCCTGGGGGACGATGAG ATT-3′	5′-AGTCGCCAGTACACAA GGTG-3′
b-actin	5′-CTCCTGAGCGCAAGTACT CTGTG-3′	5′-TAAAACGCAGCTCAGTAAC AGTCC-3′

### Electrophysiological analysis

Horizontal hippocampal slices 300 μm thick were prepared from the brains of control and stressed mice at the age of 5–7 weeks. Prior to decapitation mice were anesthetized by isoflurane. The slicing chamber contained an oxygenated ice-cold solution composed of (in mM): *K*-Gluconate, 140; *N*-(2-hydroxyethyl) piperazine-*N*’-ethanesulfonic acid (HEPES), 10; Na-Gluconate, 15; ethylene glycol-bis (2-aminoethyl)-*N, N, N*’, *N*’-tetraacetic acid, 0.2; and NaCl, 4 (pH 7.2). Slices were incubated for 30 min at 35°C before being stored at room temperature in artificial CSF (ACSF) containing (in mM): NaCl, 125; NaHCO_3_, 25; KCl, 2.5; NaH_2_PO_4_, 1.25; MgCl_2_, 1; CaCl_2_, 2; and D-glucose, 25; bubbled with 95% O_2_ and 5% CO_2_. During experiments, slices were continuously perfused with ACSF containing (in mM): NaCl, 125; NaHCO_3_, 25; KCl, 2.5; NaH_2_PO_4_, 1.25; MgCl_2_, 4; CaCl_2_, 4; and D-glucose, 25; bubbled with 95% O_2_ and 5% CO_2_. Patch electrodes were pulled from hard borosilicate capillary glass (Sutter Instruments flaming/brown micropipette puller). Electrodes for the postsynaptic pyramidal cells were filled with a solution consisting of (in mM): Cs-gluconate, 100; CsCl, 40; HEPES, 10; NaCl, 8; MgATP, 4; MgGTP, 0.3; phosphocreatine, 10 (pH 7.3 with CsOH).

CA1 pyramidal cells were visually identified using IR-video microscopy. Whole-cell recordings from these neurons were taken at room temperature (23–25°C) in voltage-clamp mode using a HEKA EPC-10 amplifier (List Elektronik) with a sampling rate of 100 μs and filtered at 3 kHz. EPSCs were evoked from two independent inputs, basal and apical dendrites, with two patch pipettes as stimulating electrodes located in *str. oriens* and *str. radiatum*, respectively (Figure 1B). The stimulus intensity was set to evoke EPSCs with an amplitude of 50–100 pA. The two stimulus pipettes were > 200 μm apart, located below and above the soma of a CA1 pyramidal cell. All measurements were at -70 mV membrane potential. In LTP experiments the control pathway was measured by stimulating synapses of the basal dendrites when apical dendrite input was potentiated and vice versa: the input in *str. radiatum* was used as a control pathway when the paired pathway was the input to synapses of the basal dendrites. LTP was evoked and recorded as described in Chen et al. (1999) by voltage clamping the membrane potential of the postsynaptic pyramidal cell to 0 mV for 3 min while stimulating the paired pathway every 1.5 s. Series resistance was monitored, and data from cells in which series resistance varied by > 15% during recording were discarded from analysis. All experiments were done in the presence of SR-95531 to block GABAA receptor channels.

### *In vivo* recording

Experiments were performed using adult Wistar rats of both sexes (250–300 g). A total of 40 rats were used in this study (*n* = 8–12 per group). Electrophysiological experiments were performed when the animals reached an age of more than 2 months. For the fEPSP recording rats were anesthetized with urethane (1.75 g/kg, intraperitoneally) and mounted in a stereotaxic frame for surgical preparation. A nickel-chrome stimulating electrode (diameter 80 μm) was implanted into the Schaffer Collaterals (SC) region of the hippocampus (3.0 mm posterior, 3.0 mm lateral to bregma, approximately 2.8 mm ventral to dura). Stimulating electrodes were inserted to a depth of 2.0 mm and gradually lowered to the required depth (but not deeper than 2.8 mm) depending on the recorded response in the CA1 region. The recording electrode was placed into the CA1 area (2.7 mm posterior, 1.5 lateral to bregma, approximately 2.2–2.5 mm ventral to dura) (Paxinos and Watson, 2007; Figure 4C). One electrode under the skin served as a ground and reference electrode.

The fEPSPs in the CA1 field were evoked by stimulation of the Schaffer collaterals (interstimulus interval 30 ms, intertrain time 30 s at intensity of 100–400 μA). After stabilization of responses, baseline was recorded for at least 30 min. To induce long-term potentiation, a theta-burst protocol was used (five trains with an interval of 30 s) Each theta-burst train consist of four bursts, each with five stimuli at a frequency of 100 Hz with 200 ms between bursts.

## Results

### GRIA and GRIN transcription transcription in control and stressed animals

Several studies have reported that ELS can alter the expression profile of glutamate receptor (GluR) channels (Ryan et al., 2009; Wilkinson et al., 2024; Lesuis et al., 2019). However, the published data are quite contradictory. Therefore, we decided to evaluate global mRNA levels in control and ELS animals (Figure 1A). No significant changes in mRNA levels encoding AMPAR subunits induced by ELS were detected in either male or female mice (Figure 1C). We found no significant effects of ELS on GRIN1 mRNA. A small but significant increase in GRIN2A transcripts was detected in ELS males, but compared to ELS females, this increase in GRIN2A mRNA was insignificant. RT-PCR analysis of GRIN2B, GRIN2C, and GRIN2D mRNA also showed no significant changes associated with ELS (Figure 1D).

### Early-life stress does not affect basic release properties at CA3 to CA1 pyramidal cell synapses and LTP at the basal dendrite inputs

**FIGURE 2:**
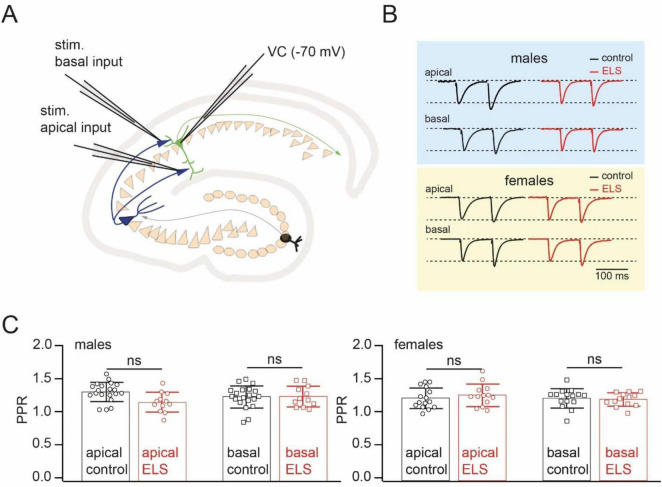
Early-life stress (ELS) has no effect on paired pules facilitation at CA3 to CA1 cell synapses. **(A)**. Schematic drawing of the experimental setup used for stimulation of spatially separated synaptic inputs to CA1 pyramidal cells. **(B)**. Example of averaged EPSCs evoked by paired-pulse stimulation (interstimulus interval 100 ms) of inputs converging on the apical and basal dendrites of CA1 pyramidal neurons in control (black) and ELS (red) mice. The amplitudes of the first EPSCs in control and ELS mice were in the range of 50–100 pA. Traces are scaled by the amplitude of the first EPSC to simplify comparison. **(C)**. The plots compare paired pulse ratios (PPR) of EPSCs in control and ELS animals evoked by stimulation of inputs to apical and basal dendrites of CA1 pyramidal cells. Data are presented as mean ± SD. The statistical significance of the differences between control and ELS data were assessed using the Student’s *t*-test; ns stands for non-significant (*p* > 0.05).

To assess possible effects on the basic synaptic release properties, short-term synaptic dynamics, and long-term synaptic efficacy changes, whole-cell voltage-clamp recordings were performed in acute horizontal hippocampal slices from control and ELS-treated mice. In each experiment, two separate inputs, on apical and basal dendrites, were stimulated with stimulus pairs via glass pipettes placed in *stratum radiatum* and *stratum oriens*, respectively (Figure 2A). Two stimuli separated by 100 ms evoked pairs of EPSCs with paired-pulse facilitation characteristic for CA3 to CA1 pyramidal cell synapses. The magnitude of facilitation, measured as the EPSP2/EPSP1 ratio, was very similar in control and stressed mice when compared across sex and synaptic input locations (Figures 2B, C).

**FIGURE 3:**
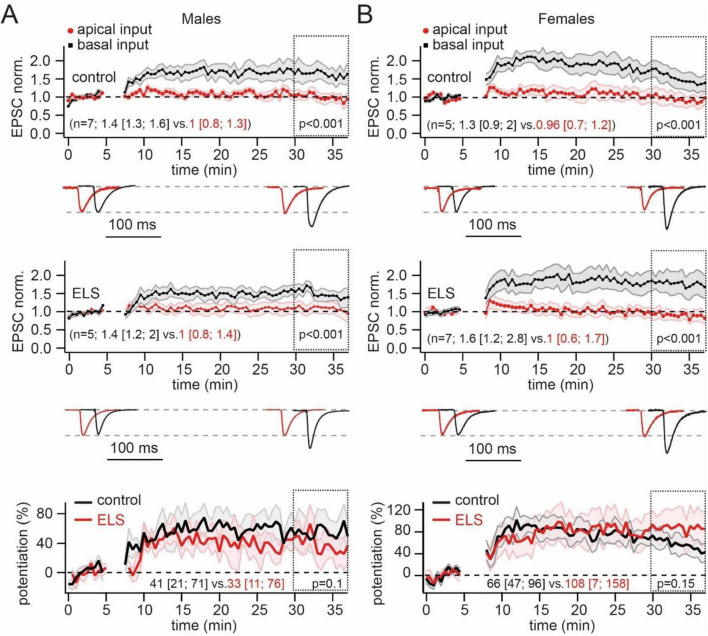
Early-life stress (ELS) mice showed no difference in the pairing-induced long-term potentiation (LTP) at basal dendrites compared to control animals. **(A)**. Pairing of neuron depolarization with *stratum oriens* stimulation significantly increased the amplitude of EPSCs at synapses located on basal dendrites (black symbols) in control (upper plot) and ELS mice (middle plot). No significant changes of EPSC amplitudes were observed at non-potentiated apical synapses (red symbols). Time course data for EPSC changes are presented as mean ± SEM. The statistical significance of differences between control and potentiated pathways was assessed for data within the 30–37 min window (doted boxes) using the Mann-Whitney Rank Sum test. Values in parentheses show the median (P_25_; P_75_) for potentiated (black) and control (red) pathways. The traces at the bottom show averaged EPSCs before and after LTP induction. The traces are scaled by EPSC amplitude before potentiation to facilitate comparison of the two pathways. The amplitudes of EPSCs before potentiation in control and ELS mice were in the range of 50–100 pA. The bottom plot shows averaged curves, showing the time course of potentiation expressed as percentages of changes in synaptic efficacy (mean ± SEM) in control (black) and ELS (red) males. The curves were obtained by subtraction of the control pathway (apical, non-potentiated input) from the potentiated pathway (basal input). The statistical significance of differences between control and ELS males was assessed using the Mann-Whitney Rank Sum test. Values in parentheses show the median (P_25_; P_75_) for control (black) and ELS (red) mice. **(B)**. The same as on **(A)** obtained for control and ELS females.

We next tested whether ELS could influence the plastic properties at CA3 to CA1 pyramidal cell synapses converging on basal dendrites. Some previous studies have reported that maternal separation can influence the functional expression of NMDARs (Ryan et al., 2009; Wilkinson et al., 2024; Lesuis et al., 2019). Therefore, we chose “pairing” as the induction protocol since it is completely dependent on NMDAR-mediated calcium influx (Chen et al., 1999). Pairing presynaptic stimulation of basal dendrite inputs with postsynaptic depolarization induced a significant enhancement of EPSC amplitudes in both control male and female mice. In the slices of stressed animals, pairing also resulted in LTP induction. Moreover, normalized values of post-induction EPSC amplitudes were similar to those observed in control groups (Figures 3A, B). To better compare absolute LTP values in control and ELS mice, we subtracted the control pathway (apical, non-potentiated input) from the potentiated pathway (basal input). Averaged curves showing the time course of potentiation expressed as percentages of changes in synaptic efficacy are shown in the lower panels of Figure 3. Averaged percentages of potentiation measured over a 30–37 min window in control and ELS males were nearly identical. The same analysis of the data obtained in control and ELS females also revealed no significant ELS-related changes in LTP at synapses located on basal dendrites.

### Early-life stress reduces LTP at *stratum radiatum* CA3 to CA1 pyramidal cell inputs

**FIGURE 4:**
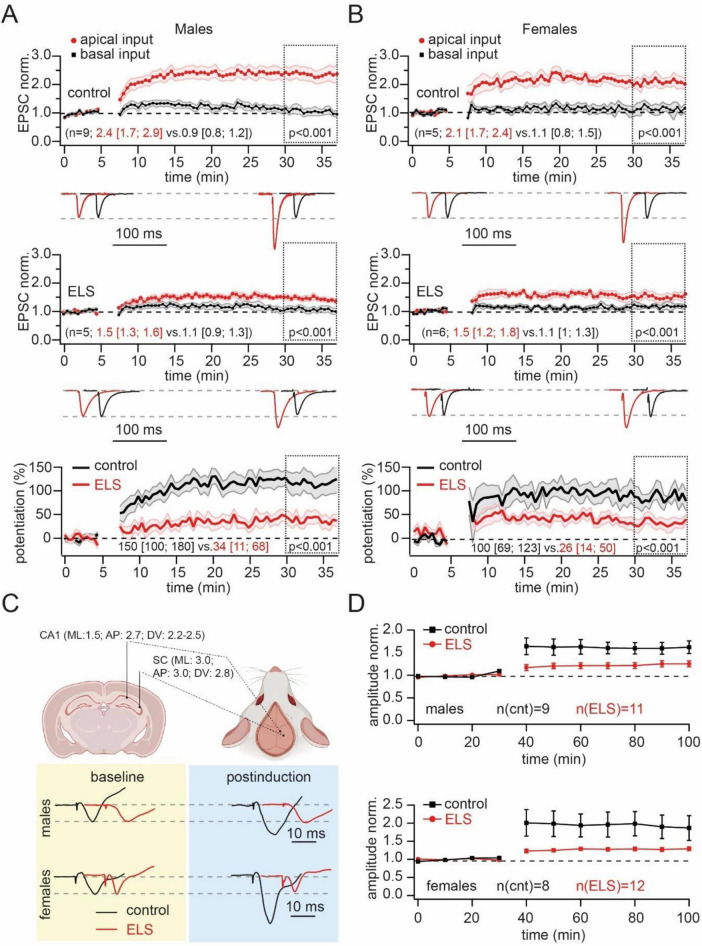
Early-life stress (ELS) strongly reduced the pairing-induced long-term potentiation (LTP) in the apical dendrites *in vitro* and *in vivo*. **(A)**. Pairing of neuron depolarization with *stratum radiatum* stimulation significantly increased the amplitude of EPSCs at synapses located on apical dendrites (red symbols) in control (upper plot) and ELS mice (middle plot). Input to basal dendrites was used as the control pathway (black symbols). Time course data for EPSC changes are presented as mean ± SEM. Statistical significance of differences between control and potentiated pathways was assessed for data within the 30–37 min window (doted boxes) using the Mann-Whitney Rank Sum test. Values in parentheses show the median (P_25_; P_75_) for potentiated (black) and control (red) pathways. The traces at the bottom show averaged EPSCs before and after LTP induction. The traces are scaled by EPSC amplitude before potentiation to facilitate comparison of the two pathways. The amplitudes of EPSCs before potentiation in control and ELS mice were in the range of 50–100 pA. The bottom plot shows averaged curves, showing the time course of potentiation expressed as percentages of changes in synaptic efficacy (mean ± SEM) in control (black) and ELS (red) males. The statistical significance of differences between control and ELS males was assessed using the Mann-Whitney Rank Sum test. Values in parentheses show the median (P_25_; P_75_) for control (black) and ELS (red) mice. Note, that the level of LTP in ELS animals was significantly smaller than in control animals. **(B)**. The same as on **(A)** obtained for control and ELS females. **(C)**. Schematic drawing shows the coordinates for placement of stimulating and recording electrodes for *in vivo* fEPSP measurements in rats. Example fEPSP traces prior to (yellow box) and post (blue box) LTP induction and in control (black) and ELS rats. The traces are scaled by fEPSP amplitude before potentiation to facilitate comparison of control and ELS rats. The amplitudes of fEPSPs before potentiation in control and ELS mice were in the range of 1–2 mV. **(D)**. The time course of fEPSP amplitudes in control (black) and ELS (red) male (upper plot) and female (bottom plot) rats. Potentiation was triggered by theta-burst stimulation. Note that while LTP in control male and female rats was statistically significant, in ELS rats of both sexes the post-induction enhancement of fEPSP amplitudes did not reach significance.

Next, we examined whether the properties of LTP at synapses converging on apical dendrites remained unchanged after separation from the mother. The pairing of presynaptic stimulation of *stratum radiatum* inputs with postsynaptic depolarization resulted in a strong and significant potentiation of apical synapses in control mice regardless of sex. In slices from ELS animals, pairing could also produce statistically significant potentiation of EPSCs. However, in both males and females, the level of potentiation was significantly reduced compared to controls. Comparison of the percentages of potentiation revealed a twofold reduction of LTP amplitude in slices of stressed animals (Figures 4A, B).

In our laboratory, we have a well-established technique for *in vivo* recordings in anesthetized rats (Dobryakova et al., 2020; Dobryakova et al., 2023). To substantiate and generalize our findings that ELS reduces LTP at excitatory synapses located on the apical dendrites of CA1 neurons, we studied the effects of ELS on *in vivo* evoked LTP in the dorsal hippocampus of adult (>2 months) Wistar rats. Experiments were performed under urethane anesthesia. Stimulating and recording electrodes were implanted as shown in Figure 4C. The fEPSPs in the CA1 field were evoked by stimulation of Schaffer collaterals. After stabilization of the responses, baseline responses were recorded for at least 30 min. LTP was induced by using a theta-burst protocol (see section “Materials and method” for details). After potentiation, fEPSPs were recorded for at least an hour. In control males and females, theta-burst stimulation produced at least a 60% increase in fEPSP amplitudes. However, in ELS animals, the average enhancement of fEPSP amplitudes were significantly lower, reaching potentiation values of less than 30% relative to baseline responses (Figure 4D). Two-way ANOVA for repeated measures did not show a significant effect of ELS on fEPSP amplitude in male rats (F 1,15 = 3.94, *p* = 0.066). However, significant differences were found for the time (F 10,150 = 12.15, *p* = 0.0001) and time and stress interaction (F 10,150 = 2.9, *p* = 0.002). Detailed *post hoc* analysis revealed that ELS induced a significant reduction of the LTP amplitude in male rats in the first 40 min after LTP induction compared with animals from the control group.

The fEPSP amplitude in ELS female rats was also significantly lower after LTP induction compared to the amplitude in control female animals (F 1,16 = 4.75, *p* = 0.045). A significant time effect (F 10,160 = 14.19, *p* < 0.01) and interaction between stress and time (F 10,160 = 4.35, *p* < 0.01) were found for the fEPSP amplitude.

In ELS males and females the amplitude of EPSPs after induction of LTP was not statistically different from the amplitude of EPSPs before potentiation.

Thus, regardless of differences in experimental approach (*in vitro* vs. *in vivo*), different animals (mice vs. rats), different recording sites (ventral vs. dorsal hippocampus), different recoding techniques and LTP induction protocols (whole cell vs. LFP recording; pairing vs. theta-burst stimulation), our data allow us to conclude that maternal separation-induced ELS reduces potentiation capacity at synapses located on apical dendrites.

## Discussion

The dendritic tree of the pyramidal neuron has two distinct domains: the basal and apical dendrites. The large apical dendrite arises from the soma toward *stratum radiatum* and arborizes distally, close to stratum lacunosun moleculare, to form the apical tuft, while the short basal dendrites arise from the base of the soma and spread into *stratum oriens*. The two domains differ in morphology, afferent connections, and ion channel distribution. In general, the distal apical dendrites of CA1 pyramidal neurons receive input from distal regions including the entorhinal cortex and the thalamic nucleus reuniens, while the basal and proximal apical dendrites receive input primarily from CA3 cells via Schaffer collaterals, with distal CA3 neurons projecting primarily to the apical dendrites and CA3 neurons closer to CA1 projecting more to the basal dendrites (Ishizuka et al., 1990; Li et al., 1994). Early in rodent life, the development of excitatory projections to different dendritic domains of CA1 pyramidal cells occurs at different times and is controlled by different input signals. Distal synapses receiving information from the entorhinal cortex develop first in late embryonic stages. Shortly after birth, these synapses are engaged into the transmission early sharp waves (eSPW), that carry sensory information generated by myoclonic movements (Cossart and Khazipov, 2022). It has been shown that eSPW are involved in the proper maturation of synapses mediating perforant path inputs to DG granule cells, CA3 and CA1 pyramidal neurons (Donato et al., 2017). Interestingly, maternal separation during the period of eSPW activity leads to enhancement of LTP at perforant path inputs to DG granule cells in adult rats (Kehoe et al., 1995; Kehoe and Bronzino, 1999; Bronzino et al., 1996). Projections from CA3–CA1 pyramidal cells begin to develop during the first postnatal week. Although published data to date are conflicting, most available studies suggest that maternal separation results in decreased LTP at CA3 to CA1 pyramidal cell synapses located on apical dendrites (Wilkinson et al., 2024; Sousa et al., 2014; Shin et al., 2016; Ryan et al., 2009; Lesuis et al., 2019; Herpfer et al., 2012). It should be noted here that maternal separation involves additional social stress caused by separation of the pups from each other. It is likely that the greater sensory deprivation associated with maternal separation explains the greater physiological impact of this ELS protocol compared to maternal deprivation. However, nothing is known about the influence of ELS on the plastic properties of CA3–CA1 pyramidal cell synapses located on basal dendrites. Several lines of evidence suggest that CA3 input to basal dendrites has a distinct function and differs from apical synapses in a number of features important for synaptic transmission and plasticity. LTP at apical dendrites can be modulated by NO, while potentiation at basal dendrite synapses seems to be NO insensitive (Ramachandran et al., 2015). Shank 2 proteins differentially affect both AMPAR expression and the magnitude of LTP in distinct lamina of hippocampal CA1 dendrites (Eltokhi et al., 2021). Finally, it has recently been suggested that a significant number of pyramidal cells have an initial axon segment originating from one of the basal dendrites, meaning that *stratum oriens* inputs may occupy a privileged position in transmission of excitation and signal processing (Stingl et al., 2024; Hodapp et al., 2022), The excitation-drive converging on axon-carrying dendrite escapes the control of perisomatic inhibition on the way to the axonal initial segment (AIS). When perisomatic inhibition is active, signal propagation from all dendritic branches to the axon is shunted, except from the axon-carrying dendrite where excitation reaches the AIS before encountering the inhibitory shunt. It was also shown that mainly those CA1 pyramidal cells whose axon emerges from the basal dendrite are able to fire during sharp wave-ripple oscillations, which suggests that in the excitatory flow in the hippocampal-entorhinal cortex loop, the CA3 input to the basal dendrites plays an exceptional role (Hodapp et al., 2022).

Although the precise mechanism by which early life stressors selectively reduce LTP at synapses located in *stratum radiatum* remains to be elucidated, our findings suggest that stressful experiences during a critical developmental period may shift the primary locus of plasticity from apical to basal dendrites. We can speculate that in animals subjected to ELS, the importance of synaptic connections from CA3 to CA1 cells in *stratum oriens* may be further enhanced, since, unlike apical inputs, these synapses retain their plastic capacity.

Thus, the published data and our findings show that maternal separation reduces the ability of neurons to undergo LTP at local intrahippocampal synapses, mossy fiber input to CA3 pyramidal cells and Shaffer collateral input to the apical domain of CA1 pyramidal neurons (Sousa et al., 2014; Shin et al., 2016; Lesuis et al., 2019; Herpfer et al., 2012). However, plastic properties at connections that are involved in maintenance of excitation flow between the hippocampus and entorhinal cortex either enhance (Kehoe et al., 1995; Kehoe and Bronzino, 1999; Bronzino et al., 1996; Blaise et al., 2008) or remain unaffected.

## Data Availability

The raw data supporting the conclusions of this article will be made available by the authors, without undue reservation.

## References

[B1] Blaise J. H. Koranda J. L. Chow U. Haines K. E. Dorward E. C. (2008). Neonatal isolation stress alters bidirectional long-term synaptic plasticity in amygdalo-hippocampal synapses in freely behaving adult rats. *Brain Res.* 1193 25–33. 10.1016/j.brainres.2007.11.049 18178177

[B2] Bronzino J. D. Kehoe P. Austin-Lafrance R. J. Rushmore R. J. Kurdian J. (1996). Neonatal isolation alters LTP in freely moving juvenile rats: Sex differences. *Brain Res. Bull.* 41 175–183. 8886387 10.1016/0361-9230(96)00166-9

[B3] Chen H. X. Otmakhov N. Lisman J. (1999). Requirements for LTP induction by pairing in hippocampal CA1 pyramidal cells. *J. Neurophysiol.* 82 526–532. 10444652 10.1152/jn.1999.82.2.526

[B4] Cossart R. Khazipov R. (2022). How development sculpts hippocampal circuits and function. *Physiol. Rev.* 102 343–378. 34280053 10.1152/physrev.00044.2020

[B5] Dobryakova Y. V. Gerasimov K. Spivak Y. S. Korotkova T. Koryagina A. Deryabina A. (2023). The induction of long-term potentiation by medial septum activation under urethane anesthesia can alter gene expression in the hippocampus. *Int. J. Mol. Sci.* 24:12970. 10.3390/ijms241612970 37629149 PMC10454684

[B6] Dobryakova Y. V. Stepanichev M. Y. Markevich V. A. Bolshakov A. P. (2020). Long-term potentiation in the hippocampal CA3 to CA1 synapses may be induced in vivo by activation of septal cholinergic inputs. *Int. J. Neurosci.* Online ahead of print. 10.1080/00207454.2020.1822834 32916077

[B7] Donato F. Jacobsen R. I. Moser M. B. Moser E. I. (2017). Stellate cells drive maturation of the entorhinal-hippocampal circuit. *Science* 355:eaai8178. 10.1126/science.aai8178 28154241

[B8] Eltokhi A. Gonzalez-Lozano M. A. Oettl L. L. Rozov A. Pitzer C. Röth R. (2021). Imbalanced post- and extrasynaptic SHANK2A functions during development affect social behavior in SHANK2-mediated neuropsychiatric disorders. *Mol. Psychiatry* 26 6482–6504. 34021263 10.1038/s41380-021-01140-y PMC8760046

[B9] Heim C. Nemeroff C. B. (2002). Neurobiology of early life stress: Clinical studies. *Semin. Clin. Neuropsychiatry* 7 147–159. 11953939 10.1053/scnp.2002.33127

[B10] Herpfer I. Hezel H. Reichardt W. Clark K. Geiger J. Gross C. M. (2012). Early life stress differentially modulates distinct forms of brain plasticity in young and adult mice. *PLoS One* 7:e46004. 10.1371/journal.pone.0046004 23071534 PMC3465301

[B11] Hodapp A. Kaiser M. E. Thome C. Ding L. Rozov A. Klumpp M. (2022). Dendritic axon origin enables information gating by perisomatic inhibition in pyramidal neurons. *Science* 377 1448–1452. 36137045 10.1126/science.abj1861

[B12] Imbriglio T. Verhaeghe R. Antenucci N. Maccari S. Battaglia G. Nicoletti F. (2021). Developmental up-regulation of NMDA receptors in the prefrontal cortex and hippocampus of mGlu5 receptor knock-out mice. *Mol. Brain* 14:77. 10.1186/s13041-021-00784-9 PMC8106212 33962661

[B13] Ishizuka N. Weber J. Amaral D. G. (1990). Organization of intrahippocampal projections originating from CA3 pyramidal cells in the rat. *J. Comp. Neurol.* 295 580–623. 10.1002/cne.902950407 2358523

[B14] Kehoe P. Bronzino J. D. (1999). Neonatal stress alters LTP in freely moving male and female adult rats. *Hippocampus* 9 651–658. 10641758 10.1002/(SICI)1098-1063(1999)9:6<651::AID-HIPO6>3.0.CO;2-P

[B15] Kehoe P. Hoffman J. H. Austin-Lafrance R. J. Bronzino J. D. (1995). Neonatal isolation enhances hippocampal dentate response to tetanization in freely moving juvenile male rats. *Exp. Neurol.* 136 89–97. 7498418 10.1006/exnr.1995.1086

[B16] Lesuis S. L. Lucassen P. J. Krugers H. J. (2019). Early life stress impairs fear memory and synaptic plasticity; A potential role for GluN2B. *Neuropharmacology* 149 195–203. 10.1016/j.neuropharm.2019.01.010 30641077

[B17] Li X. G. Somogyi P. Ylinen A. Buzsáki G. (1994). The hippocampal CA3 network: An in vivo intracellular labeling study. *J. Comp. Neurol.* 339 181–208. 10.1002/cne.903390204 8300905

[B18] Oomen C. A. Soeters H. Audureau N. Vermunt L. van Hasselt F. N. Manders E. M. (2011). Early maternal deprivation affects dentate gyrus structure and emotional learning in adult female rats. *Psychopharmacology (Berl)* 214 249–260. 20589492 10.1007/s00213-010-1922-8 PMC3045507

[B19] Oomen C. A. Soeters H. Audureau N. Vermunt L. van Hasselt F. N. Manders E. M. (2010). Severe early life stress hampers spatial learning and neurogenesis, but improves hippocampal synaptic plasticity and emotional learning under high-stress conditions in adulthood. *J. Neurosci.* 30 6635–6645. 10.1523/JNEUROSCI.0247-10.2010 20463226 PMC6632559

[B20] Paxinos G. Watson C. (2007). *The rat brain in stereotaxic coordinates*. Amsterdam: Elsevier. 10.1016/0165-0270(80)90021-7 6110810

[B21] Priya A. Johar K. Nair B. Wong-Riley M. T. (2014). Specificity protein 4 (Sp4) regulates the transcription of AMPA receptor subunit GluA2 (Gria2). *Biochim. Biophys. Acta* 1843 1196–1206. 24576410 10.1016/j.bbamcr.2014.02.008 PMC4024218

[B22] Ramachandran B. Ahmed S. Dean C. (2015). Long-term depression is differentially expressed in distinct lamina of hippocampal CA1 dendrites. *Front. Cell Neurosci.* 9:23. 10.3389/fncel.2015.00023 25767434 PMC4341561

[B23] Ryan B. Musazzi L. Mallei A. Tardito D. Gruber S. H. El Khoury A. (2009). Remodelling by early-life stress of NMDA receptor-dependent synaptic plasticity in a gene-environment rat model of depression. *Int. J. Neuropsychopharmacol.* 12 553–559. 10.1017/S1461145708009607 18976544

[B24] Shin S. Y. Han S. H. Woo R. S. Jang S. H. Min S. S. (2016). Adolescent mice show anxiety- and aggressive-like behavior and the reduction of long-term potentiation in mossy fiber-CA3 synapses after neonatal maternal separation. *Neuroscience* 316 221–231. 10.1016/j.neuroscience.2015.12.041 26733385

[B25] Sousa V. C. Vital J. Costenla A. R. Batalha V. L. Sebastião A. M. Ribeiro J. A. (2014). Maternal separation impairs long term-potentiation in CA1-CA3 synapses and hippocampal-dependent memory in old rats. *Neurobiol. Aging* 35 1680–1685. 10.1016/j.neurobiolaging.2014.01.024 24559649

[B26] Stingl M. Draguhn A. Both M. (2024). A dendrite is a dendrite is a dendrite? Dendritic signal integration beyond the “antenna” model. *Pflugers Arch.* 477 9–16. 10.1007/s00424-024-03004-0 39162833 PMC11711151

[B27] Thome C. Kelly T. Yanez A. Schultz C. Engelhardt M. Cambridge S. B. (2014). Axon-carrying dendrites convey privileged synaptic input in hippocampal neurons. *Neuron* 83 1418–1430. 10.1016/j.neuron.2014.08.013 25199704

[B28] Wilkinson M. P. Robinson E. S. J. Mellor J. R. (2024). Analysis of hippocampal synaptic function in a rodent model of early life stress. *Wellcome Open Res.* 9:300. 10.12688/wellcomeopenres.22276.1 39221440 PMC11362746

